# Grouping Influences Output Interference in Short-term Memory: A Mixture Modeling Study

**DOI:** 10.3389/fpsyg.2016.00585

**Published:** 2016-05-02

**Authors:** Min-Suk Kang, Byung-Il Oh

**Affiliations:** ^1^Center for Neuroscience and Imaging Research, Institute for Basic ScienceSuwon, South Korea; ^2^Department of Psychology, Sungkyunkwan UniversitySeoul, South Korea

**Keywords:** output interference, grouping, short-term memory, mixture modeling

## Abstract

Output interference is a source of forgetting induced by recalling. We investigated how grouping influences output interference in short-term memory. In Experiment 1, the participants were asked to remember four colored items. Those items were grouped by temporal coincidence as well as spatial alignment: two items were presented in the first memory array and two were presented in the second, and the items in both arrays were either vertically or horizontally aligned as well. The participants then performed two recall tasks in sequence by selecting a color presented at a cued location from a color wheel. In the same-group condition, the participants reported both items from the same memory array; however, in the different-group condition, the participants reported one item from each memory array. We analyzed participant responses with a mixture model, which yielded two measures: guess rate and precision of recalled memories. The guess rate in the second recall was higher for the different-group condition than for the same-group condition; however, the memory precisions obtained for both conditions were similarly degraded in the second recall. In Experiment 2, we varied the probability of the same- and different-group conditions with a ratio of 3 to 7. We expected output interference to be higher in the same-group condition than in the different-group condition. This is because items of the other group are more likely to be probed in the second recall phase and, thus, protecting those items during the first recall phase leads to a better performance. Nevertheless, the same pattern of results was robustly reproduced, suggesting grouping shields the grouped items from output interference because of the secured accessibility. We discussed how grouping influences output interference.

## Introduction

Output interference is a type of forgetting induced by recalling (Tulving and Arbuckle, 1966; Roediger, 1974; Nickerson, 1984; Anderson et al., 1994). One of the earliest studies of the output interference was conducted by Tulving and Arbuckle (1966), who asked their participants to remember a list of word and number pairs. They then provided a word cue to aid in the recall of the paired number. Participants’ memory performance decreased with an increase in the number of items to be recalled. The retrieval-practice paradigm shows another type of forgetting induced by recalling (Anderson et al., 1994; Anderson and Spellman, 1995; Anderson, 2003). The participants in these studies learned lists of category and exemplar pairs (e.g., Fruit: Orange, Fruit: Apple, Animal: Elephant, and Animal: Lion), and then they practiced retrieving a subset of exemplars with the associated category cue (e.g., Fruit: Or____). They recalled many of the practiced exemplars (e.g., Orange). Surprisingly, they recalled more unpracticed exemplars from the unpracticed categories (e.g., Elephant) than they did the unpracticed exemplars of the practiced categories (e.g., Apple). This specific memory impairment for the unpracticed exemplars of the practiced category compared to the unpracticed category is the hallmark outcome of the retrieval practice paradigm and has been discussed in terms of retrieval-induced forgetting. Levy and Anderson (2002) and Anderson (2003) proposed that retrieval-induced forgetting occurs because memory retrieval inhibits competing representations that share the same cue. Kang and Choi (2015) demonstrated that memory retrieval inhibits competing representations, even when participants remember only two motion directions. This is convincing evidence for the inhibition account considering that our short-term memory capacity is larger than two.

In the present study, we asked whether grouping influences output interference as grouping facilitates our memory (Wheeler and Treisman, 2002; Xu and Chun, 2006; Luria et al., 2010; Luria and Vogel, 2011). In particular, we intended to distinguish two types of output interference: disruption in memory precision from disruption in memory accessibility. Two lines of studies suggest grouping would protect the grouped items from output interference by securing accessibility.

First, previous studies have suggested that grouping protects items from output interference. Cowan et al. (2002), the participants were serially presented with nine digits at 1-s intervals, and they had to recall as many digits and their input positions as possible during the retrieval phase. In the grouped list condition, an additional 1-s interval was given after the third and sixth digit presentations, resulting in three groups of three digits each. They found that participants remembered the list better in the grouped condition than in the ungrouped condition, especially when the list was presented acoustically. Anderson and McCulloch (1999) asked a similar question using a retrieval-practice paradigm. In a control condition, they gave their participants category and exemplar pairs (e.g., Fruit: Orange, Fruit: Apple) and asked them to study those pairs without further instruction. In an integrative-rehearsal condition, the participants were additionally instructed to rehearse each item with previously studied items from the same category. They found that those participants assigned to the integrative-rehearsal condition remembered more items after the retrieval-practice phase than did the control condition participants. They obtained a similar result, even when extra study time was given to those in the control condition, indicating that integrated memory representations in memory are resistant to the forgetting induced by memory retrieval.

Second, memory retrieval of an item appears to disrupt the accessibility to a greater extent than does the precision of the other items being held in memory. When participants remembered a list of several categories and category members, their performance of a cued recall task was superior to their performance of a free recall task. This indicated that they could not access the memory representations in the free recall task, even though the memory representations were available, as evidenced by the superior cued recall performance (Tulving and Pearlstone, 1966). MacLeod and Macrae (2001) arrived at a similar conclusion. If their participants’ memories were probed immediately after a retrieval-practice phase, retrieval-induced forgetting occurred. However, if the participants were tested 24 h later, the retrieval-induced forgetting did not occur. If memory retrieval disrupted the accessibility along with the linked memory representation being held in memory, the retrieval-induced forgetting should persist even 24 h later. In a related vein, Sutterer and Awh (2015) have shown that practicing memory retrieval enhances memory accessibility, but not its precision.

**FIGURE 1:**
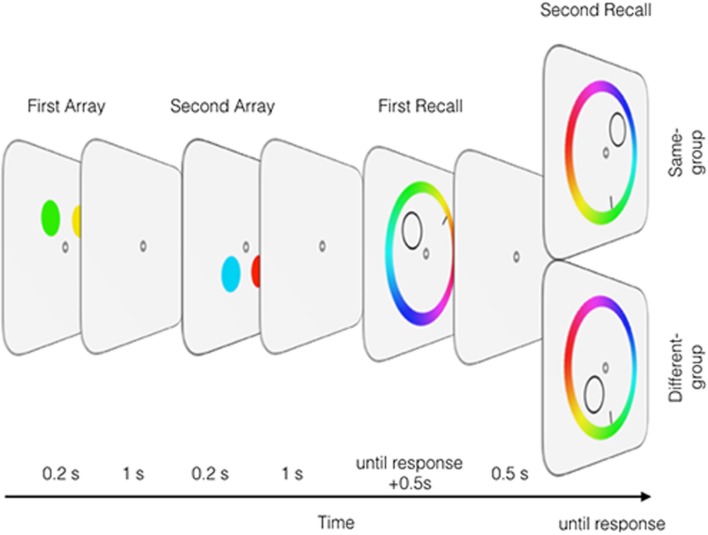
**An illustration of Experiments 1 and 2.** The participants remembered four colors presented over two memory arrays and then performed two recall tasks by selecting the remembered color at the cued location from a continuously varying color wheel. In the same-group condition, two items of the same memory array were cued, while in the different-group condition one item was selected from the first and the other selected from the second array.

In this study, we asked whether grouping influences output interference and we reasoned that mixture modeling analysis in conjunction with a continuous recall paradigm should be useful for distinguishing disruption in memory precision from disruption in memory accessibility (Wilken and Ma, 2004; Bays and Husain, 2008; Zhang and Luck, 2008). We first describe the experimental procedure, and then provide a rationale for distinguishing those two types of output interference using mixture modeling. **Figure 1** illustrates a stimulus sequence in which two colored items are presented in the first memory array, and two more colored items are presented in the second memory array. Because of the temporal coincidence of the two colors and their spatial alignments (vertical or horizontal), the two items in each memory array were assumed to be grouped during the encoding phase. Note that we do not distinguish which grouping factor plays a more dominant role; however, considering the participants had to remember only four items within a 2 × 2 square grid, any configuration would have promoted a spatial grouping. Critically, two retrieval conditions were run following the two memory array presentations based on a location cue at the position of the memory item. In the same-group condition, two items from the same memory array were cued for memory recall in sequence. In the different-group condition, a single item was selected from each memory array, and then those two items were cued for memory recall in sequence.

In each recall phase, the participants were asked to remember the color of the cued item by selecting a color from a color wheel. The responses that were associated with the true color of the cued item were modeled using a von Mises distribution (the circular analog of a Gaussian distribution), which provides a measure of memory precision in terms of standard deviation. On the other hand, the participants would respond randomly over the entire color wheel if they could not encode the cued item, or if the cued item itself or a link between the cue and memory representation was disrupted. In addition, this random selection would occur if the participants reported an incorrect item among the items in the memory array because the memory representation and a link to that representation were incorrectly coupled. This response should also be uniformly distributed over the color space^1^. These random errors were modeled with a uniform distribution, whose area indicates the guess rate, provides a measure of memory accessibility.

## Experiment 1

### Materials and Methods

Twenty participants (11 females, age range: 19–28 years, mean 23.4 ± 2.3 years), including the second author, provided their informed consent before participating. This study was approved by the Sungkyunkwan University Institutional Review Board, and all the participants’ data were included in the analyses. All participants declared either normal or corrected-to-normal visual acuity.

We used Psychophysics Toolbox (Brainard, 1997) to present all the stimuli and control the experiment. The experiment booth was dimly illuminated and contained a Mac Mini (Cupertino, CA, USA) coupled with a 21-inch CRT monitor (43 cm × 39 cm in size; 1024 H × 768 V in resolution and 85 Hz in refreshing rate) with a 70-cm viewing distance.

The stimuli were four colored circles (0.2° in radius) presented over an imaginary 2 × 2 square grid and separated from the fixation point to the center of the circle by 0.8°. The color of each circle was chosen from one of 360 different colors along a color wheel created by MemToolBox (Suchow et al., 2013).

**Figure 1** shows the stimulus sequence. Each trial started when the participant pressed a spacebar. A fixation point was presented for 1 s, and then the first and second memory arrays were presented for 200 ms each, with a 1-s interval between them. Two circles consisted of a single memory array, and both arrays in each trial were either horizontally or vertically aligned with equal probability. The four stimuli of the two memory arrays occupied different parts of a 2 × 2 square grid; thus, no two circles occupied the same position. One second after the second memory array offset, two memory recall tasks were administered. In each recall task, a monochromatic circular contour (0.2° in radius) was presented as a cue at the position of one memory item alongside a color wheel (radius = 1.6° and width = 0.15°). Because all four colors were presented at different locations, a location cue was uniquely associated with a single memory item. In the color wheel, all the possible colors were presented according to their hues in a counterclockwise direction. The color wheel was rotated randomly in each trial. Participants rotated a small bar (0.24°) by moving a computer mouse either left or right, and then they clicked the mouse’s button to select a color in the color wheel to indicate the remembered color. The screen remained unchanged for 500 ms, and then the fixation point was presented for 500 ms before the second recall task began.

Two conditions were run in this experiment. In the same-group condition, two items from the same memory array were cued for memory recall in sequence. In the different-group condition, a single item was selected from each memory array, and then those two items were cued for memory recall in sequence. The first cued item was selected either from the first or second memory array with equal probability, and those two conditions were randomly presented with equal probability. Each participant completed 256 trials in total.

Response errors were modeled using a mixture of a von Mises and a uniform distribution, in which the former was a model for memory precision and the latter was a model for the several types of guesses mentioned above (Zhang and Luck, 2008). The mixture model parameters were estimated using the MemToolBox (Suchow et al., 2013).

### Results

First, we examined the serial position effect of the first recall task. The standard deviation σ was lower if the item belonged to the second memory array (20.75 ± 3.70°) than the first one (22.88 ± 3.39°) and the difference was significant [*t*(19) = 2.78, *p* = 0.012]. The guess rate shows similar numerical patterns for the first (11.26 ± 7.09%) and second (9.73 ± 6.41%) input positions, but the difference between them is not statistically significant [*t*(19) = 1.36*, p* = 0.190]. These results are consistent with a previous study in that participants’ memory performance is higher for the items encoded later than for the items encoded earlier (Gorgoraptis et al., 2011). We found similar effects in Experiment 2; however, this serial position effect of the first recall will not be discussed further because it is not the focus of the present paper.

**FIGURE 2:**
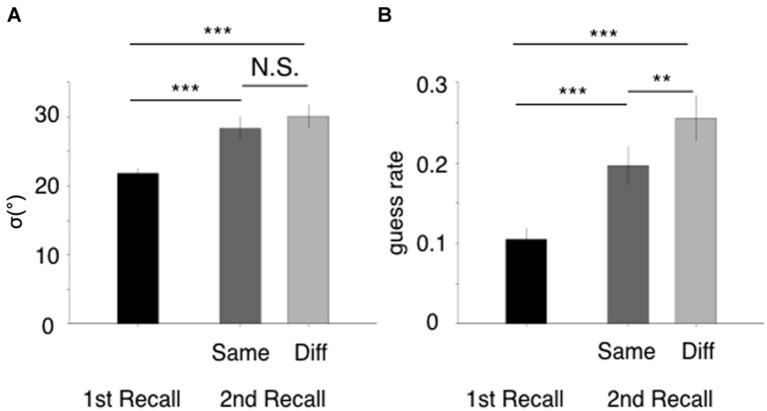
**Results of Experiment 1. (A)** Estimated standard deviations from the first recall task (black), the second recall task’s same-group condition (dark gray), and the second recall task’s different-group condition (light gray). ±1 SE are used for the error bars. Statistical significance levels obtained from pairwise comparisons are represented as N.S. for *p* > 0.1, ^∗^*p* < 0.05, ^∗∗^*p* < 0.01, ^∗∗∗^*p* < 0.001. **(B)** Estimated guess rates for the three conditions. The measurement and analysis aspects are the same as those presented for **(A)**.

**Figure 2** shows participants’ recall performance for the first and second retrievals, showing that the precision and the guess rates exhibit different result patterns for the same- and different-group conditions. Specifically, **Figure 2A** shows the standard deviations obtained from the first recall (black), the same-group condition’s second recall (dark gray), and the different-group condition’s second recall (light gray). A one-way ANOVA yielded a significant main effect among these three retrieval conditions [*F*(2,38) = 16.19, *p* < 0.001]. We also performed pairwise comparisons of means with Tukey contrasts, in which the *p*-values were adjusted using the Holm-Bonferroni correction method. The results of these tests indicated that the retrieval condition effect was driven by the difference between the first and second recalls, while the same- and different-group conditions were similar (same/second – first: *z* = 4.26, *p* < 0.001; different/second – first: *z* = 5.40, *p* < 0.001; same/second – different/second: *z* = 1.14, *p* = 0.254). On the other hand, all three conditions were different in terms of guess rates (**Figure 2B**). A one-way ANOVA yielded a significant main effect of retrieval conditions [*F*(2,38) = 28.39, *p* < 0.001], and all three pairwise comparisons were significant (same/second – first: *z* = 4.56, *p* < 0.001; different/second – first: *z* = 7.48, *p* < 0.001; same/second – different/second: *z* = 2.92, *p* = 0.003). These results indicate that the act of recalling in the first recall task is a source of forgetting because both measures increased along with the output positions. More importantly, the guess rate was lower in the same-group condition than in the different-group condition, while the memory precisions for both conditions were similar, indicating that grouped items were shielded from output interference.

## Experiment 2

The guess rate was higher in the different-group condition than in the same-group condition; however, the representation qualities were similarly degraded for both the conditions. In Experiment 2, we tested the result more thoroughly by explicitly manipulating the participants’ strategies.

We varied the probabilities of the same- and different-group conditions with a ratio of 3 to 7. In response to this manipulation, the participants should protect the other group’s items in the first recall phase because those items are more likely to be probed in the second recall. As a result, we expected output interference to be higher in the same-group condition. The probability manipulation was also thoroughly explained to the participants when they received their instructions.

### Materials and Methods

Twenty participants (14 females, age range: 19–28 years, mean 23.4 ± 2.6 years), including the second author, provided their informed consent before participating. This study was approved by the Sungkyunkwan University Institutional Review Board. All participants declared either normal or corrected-to-normal visual acuity. One participant’s data was excluded from the analysis because the estimated parameters of the same-group condition were unstable, varying from 29.7 to 226.6° in standard deviation and from 0.45 to 0.75 in guess rate.

All aspects of this experiment were identical to those of Experiment 1, except for the following two modifications. First, the different-group condition accounted for 70% of the total (224) trials, while the same-group condition made up only 30% of the total (96) trials. Second, feedback was given via an auditory signal and the visual presentation of a red bar (0.24°) at the cued color’s position if a response error was greater than ±30° from the actual memory item (25.39 ± 8.13% of the trials). Even though we provided feedback during the experiments, all these trials were included for the analysis.

### Results

**FIGURE 3:**
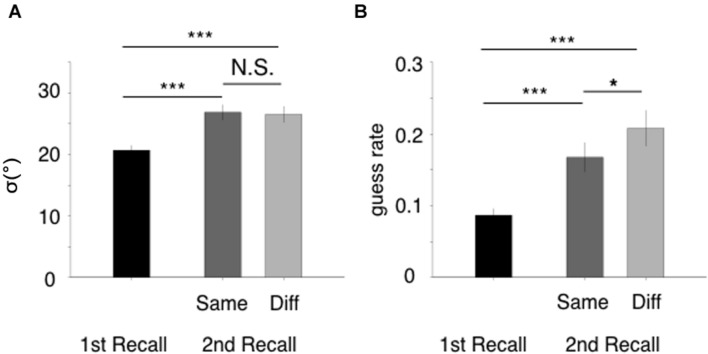
**Results of Experiment 2.** Explanatory details are identical to those for **Figure 2**.

The different-group condition was presented more frequently than the same-group condition (7:3); thus, it was reasonable to assume that the performance of the different-group condition would be superior to that of the same group-condition. However, we found the same pattern of results as in Experiment 1 (**Figure 3**). Specifically, a one-way ANOVA with a factor of the three retrieval conditions yielded a significant difference in the standard deviation [**Figure 3A**, *F*(2,36) = 19.87, *p* < 0.001], which was driven by the difference between the first and second recalls (second/same – first: *z* = 5.61, *p* < 0.001; second/different – first: *z* = 5.29, *p* < 0.001; second/same – second/different: *z* = 0.32, *p* = 0.75). We also found a significant effect of retrieval condition for the guess rate [**Figure 3B**, *F*(2,36) = 23.28, *p* < 0.001], in which all three pairs were significantly different from one another (second/same – first: *z* = 4.46, *p* < 0.001; second/different – first: *z* = 6.70, *p* < 0.001; second/same – second/different: *z* = 2.25, *p* = 0.025).

## Discussion

We investigated whether grouping reduces output interference with a particular focus on whether output interference compromises memory accessibility or precision. We distinguished the two types of forgetting using a mixture model analysis. Our results show that the guess rate was higher when retrieving items from different groups compared to from the same group, while memory precision was similarly disrupted between those two conditions, suggesting that accessibility was disrupted to a greater extent in the different-group condition due to memory retrieval.

How does the grouping shield output interference by securing memory accessibility? Mediated retrieval provides a reasonable account. Paired-associate learning between a nonsense syllable and a word (e.g., Lov-Bread) facilitates paired-associate learning between the same nonsense syllable and another word (e.g., Lov-Butter) if those paired words (e.g., Bread-Butter) are associated also. Verbal mediation accounts for this facilitation by positing indirect retrieval routes (e.g., Lov-Bread-Butter) among competing associations (Jenkins, 1963; Kjeldergaard, 1968). Anderson and McCulloch (1999) also suggested that mediation is responsible for reduced retrieval-induced forgetting among integrated items. Similar mediation can account for our results. The memory retrieval of the first item disrupts a link between a cue (e.g., stimulus location) and an associated second target item (e.g., color). This means that whether items are grouped or not, the memory retrieval of the second item should be less accessible via the corresponding location cue. Nevertheless, the second item may still be accessible via an indirect route if it is grouped with the first item, because the first item operates as a cue for the second item.

Furthermore, we believe that spread of attention possibly mediates the retrieval of grouped items in our study. Object file theory posits that perceptual grouping reflects our inability to selectively attend to an individual item (Kahneman et al., 1992; Yantis, 1992) and attention spreads over grouped items (Egly et al., 1994; Vecera and Farah, 1994). Consistently, grouping facilitates transfer of perceptual representations of grouped items into visual short-term memory (Woodman et al., 2003). Specifically, when an attention-capturing cue was presented at the location of one memory item, items that were grouped with the cued item tended to be stored in visual short-term memory. In the context of the present study, even though a link between a location cue and a second target is disrupted, one can still access to the first target via an indirect route formed by the spread of attention from the first recalled item to the second target.

## Author Contributions

M-SK designed the experiments. B-IO conducted the experiment and analyzed the data. M-SK and B-IO wrote the paper.

## Conflict of Interest Statement

The authors declare that the research was conducted in the absence of any commercial or financial relationships that could be construed as a potential conflict of interest. The reviewer HL and handling Editor declared their shared affiliation, and the handling Editor states that the process nevertheless met the standards of a fair and objective review.

## Footnotes

1 Swap errors, which are response errors that accompany the reporting of incorrect items, can be modeled with additional Gaussian distributions (Bays et al., 2011).However, we did not save relevant information for analyzing swap errors because we did not realize this possibility until we received a review of our manuscript.
